# Associated factors to the consumption of ultra-processed foods and its relation with dietary sources in Portugal

**DOI:** 10.1017/jns.2021.61

**Published:** 2021-10-07

**Authors:** Vânia Magalhães, Milton Severo, Daniela Correia, Duarte Torres, Renata Costa de Miranda, Fernanda Rauber, Renata Levy, Sara Rodrigues, Carla Lopes

**Affiliations:** 1 EPIUnit – Instituto de Saúde Pública da Universidade do Porto, Rua das Taipas, n° 135, 4050-600 Porto, Portugal; 2 Departamento de Ciências da Saúde Pública e Forenses e Educação Médica, Faculdade de Medicina da Universidade do Porto, Alameda Professor Hernâni Monteiro, 4200-319 Porto, Portugal; 3 Faculdade de Ciências da Nutrição e Alimentação da Universidade do Porto, Rua do Campo Alegre, n° 823, 4150-180 Porto, Portugal; 4 Núcleo de Pesquisas Epidemiológicas em Nutrição e Saúde, Universidade de São Paulo, São Paulo 01246-904, Brazil; 5 Departamento de Medicina Preventiva, Escola de Medicina, Universidade de São Paulo, São Paulo 01246-903, Brazil; 6 Departamento de Nutrição, Escola de Saúde Pública, Universidade de São Paulo, São Paulo 01246-904, Brazil

**Keywords:** National survey, Nova, Portugal, Ultra-processed foods

## Abstract

Ultra-processed foods (UPFs) are common worldwide and associated with poorer health outcomes. This work aimed to explore the UPF consumption associated factors and its main dietary sources, by sex, in Portugal. Participants from the National Food, Nutrition and Physical Activity Survey (IAN-AF) 2015–2016, aged 3–84 years, were included (*n* 5005). Dietary intake was assessed through two 1-day food diaries/24 h recalls. UPFs were identified using the NOVA classification. Associations were evaluated through linear regression models. Median UPF consumption was 257 g/d (10⋅6 % of total quantity; 23⋅8 % of total energy). Adolescents were those with higher consumption (490 g/d). Compared to adults, younger ages were positively associated with UPF consumption (e.g. adolescents (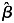-females: 192, 95 % confidence interval (CI): 135, 249; 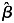-males: 327, 95 % CI: 277, 377)). A lower educational level was associated with lower UPF consumption (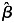-females: −63; 95 % CI: −91, −34; 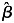-males: −68; 95 % CI: −124, −12). Also, a lower UPF consumption was observed in married males/couples compared to singles (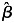: −48, 95 % CI: −96, −1). Furthermore, female current/former smokers were associated with a higher UPF consumption *v.* never smokers (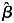: 79, 95 % CI: 41, 118; 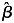: 42, 95 % CI: 8, 75, respectively). Main UPF sources were yoghurts, soft drinks and cold meats/sausages differing strongly by sex, age and education level. Yoghurts containing additives were the main contributors to the UPF consumption in children and adult females from all education (~20 %). Soft drinks were leaders in adolescents (females: 26⋅0 %; males: 31⋅6 %) and young male adults (24⋅4 %). Cold meats/sausages stood out among low-educated males (20⋅5 %). Males, younger age groups, higher education, children with less-educated parents, married/couple males and smoking females were positively associated with UPF consumption.

## Introduction

In the recent years, global changes in eating patterns have been observed, notably the increase of ultra-processed foods (UPF) consumption^(1,2)^. According to Monteiro *et al.*, the UPF manufacture includes the fractioning of whole foods into substances, chemical modifications of these substances, assembly of unmodified and modified food substances, frequent use of cosmetic additives and sophisticated packaging^(3)^. The appearance and growth of these products were due to both economic and social pressure. Ready-to-eat and ready-to-heat food products have become attractive options as societies become more urbanised, incomes have increased and the proportion of women employed outside the home has increased^(4)^. The fast movement is consolidated as a consequence of the scarcity of time^(5)^ and the globalised world favours the mass production and consumption of convenience foods. Globally, bakery products (such as cakes, sweets and industrial breads) and soft drinks have been identified as the main contributors to the UPF sales volume^(6)^. However, other food groups are relevant depending on the region, such as dairy products, processed fruits and vegetables, or baked goods^(6)^.

Data from the US National Health and Nutrition Examination Survey 2011–2016^(7)^ showed that UPF contributed to 55⋅4 % of energy intake in adults. Similar results were found in the UK National Diet and Nutrition Survey 2008–2014 describing 56⋅8 % UPF contribution to total energy intake for total population^(8)^. Moreover, a recent study in Portuguese adults and elderly^(9)^ found a dietary share of 23⋅8 and 16⋅0 %, respectively. This study has also shown that as UPF consumption increases, energy and carbohydrate intake also increases, while fibre, sodium and potassium intake decreases. The negative impact of UPF consumption on health has been systematically reviewed. Its high consumption has already been associated with overweight and obesity^(10–13)^, other cardio metabolic risk-related outcomes^(12–14)^, some types of cancer, depression, frailty^(13)^ and even mortality^(11)^.

To better support the development of food policies adapted to the needs of each population, it is important to identify which factors are associated with UPF consumption and which are its main dietary sources. Worldwide, few studies have assessed the association between sociodemographic and behavioural factors and UPF consumption. None, to our knowledge, has assessed its relationship with its main dietary sources. Studies in high-income countries have reported an inverse association between the consumption of UPF and markers of socio-economic position,^(5,15,16)^ but the opposite has been reported in Brazil^(17)^ and Colombia^(18)^. In Portugal^(19)^, compared to those having a lower socio-economic status, children and adolescents belonging to a higher socio-economic status had a higher daily intake of fruit and vegetables, white meat, fish and eggs, with no association being observed for sweets and soft drinks. However, a positive association between socio-economic status and the consumption of salty snacks was found only in adolescents, which reinforce the interest in studying UPF consumption in each age group. Moreover, the aggregation of unhealthy behaviours has been described, so that the coexistence of high UPF consumption with other unhealthy behaviours deserves to be studied.

In UK adults, similar UPF consumption was observed in subjects with different physical activity levels^(20)^. However, among Brazilian adolescents, longer time spent in sedentary behaviours was associated with a higher prevalence of UPF consumption emphasising the need for integrated health interventions^(21)^. Thus, the aim of the present study was to explore the associated sociodemographic and behavioural factors as well as the main dietary sources of UPF consumption in Portugal, by sex, using individual dietary data from a national survey. Sociodemographic factors including age, education, region, urbanisation level, civil status, household members and food insecurity were considered, while behavioural factors included physical activity and smoking status.

## Methodology

### Survey design and participants

Data from the National Food, Nutrition and Physical Activity Survey, IAN-AF, 2015–2016, were used. Details on the study design have been published previously^(22,23)^. A representative sample of the Portuguese general population, aged between 3 months and 84 years of age, was selected from the National Health Registry, by multistage sampling, in each geographical region (NUT II). The study was approved by the National Commission for Data Protection, the Ethical Committee of the Institute of Public Health of the University of Porto and from the Ethical Commissions of each of the Regional Administrations of Health. A written informed consent was obtained from all participants. For children, the informed consent was signed by parents or legal caregivers. A total of 5811 participants completed two computer-assisted face-to-face interviews conducted by trained nutritionists at the primary health care units or participants’ homes. The response rate among eligible participants was 35⋅0 %, higher in children and adolescents (approximately 46 %) and lower in the elderly (approximately 20 %). Assuming strong differences in food patterns, in the present study, participants aged less than 3 years were excluded, and the final sample was left with 5005 participants.

### Data collection

The data collection followed the guidelines of the pan-European food consumption survey (EU-Menu), previously described^(24)^. The information was collected by the interviewer using face-to-face questionnaires and directly handled in the You eAt&Move, an e-platform specifically designed for the IAN-AF^(23,25)^.

### Dietary variables

To account for seasonal variability, fieldwork was carried out within 12 months. Dietary intake was obtained by two non-consecutive food diaries in the case of children under the age of 10 years, or by two 24 h dietary recalls (8–15 days apart) for the remaining age groups^(26)^. The eAT24 module from the e-platform integrates the harmonised food classification and description system EFSA FoodEx2^(27)^ and the Portuguese food composition table^(28)^ complemented with nutritional data from other countries (1777 food items). Foods were grouped into fifteen food groups and seventy-five sub-groups, according to nutritional similarities, source and dietary use^(29)^. The eAT24 also includes several food quantification methods, such as an electronic picture book^(30)^, household measures, standard units, volume, weight and default portion. As a quality control procedure, at the end of each interview, energy and nutrient estimates were briefly analysed by the interviewer. Dietary data collected using the eAT24 software were previously validated in a subsample using urinary biomarkers^(25)^ – Pearson's correlation coefficients for protein, potassium and sodium were 0⋅33, 0⋅64 and 0⋅26, respectively.

### Food classification according to the processing degree and purpose

All reported items were classed according to the NOVA classification^(3,31)^. NOVA is a food classification system based on the degree and purpose of food processing developed by researchers at the University of São Paulo, Brazil. It classifies all foods into four groups: (1) unprocessed and minimally processed foods, (2) processed culinary ingredients, (3) processed foods and (4) UPF.

The detailed information on food intake obtained in the dietary assessment method used made it possible to classify foods from the same food group differently according to the specific characteristics of each. For example, unsweetened plain yoghurts were classified as 1, plain yoghurts with added sugar as 3 and flavoured yoghurts or with other cosmetic additives as 4.

In order to classify at the food level, recipes were previously disaggregated into ingredients. Capsule-type supplements were not included in this classification. Coding according to the degree and purpose of food processing was conducted independently by two researchers (from Portugal and Brazil). Subsequently, both lists were verified by a third researcher who identified discrepant items, later discussed among all teams and classified by consensus. In case of doubtful classification, the experts decided on the most conservative classification, the one corresponding to the lowest processing level.

### Non-dietary variables

Data on sociodemographic characteristics, food security and health behaviours were collected. Based on the address of the participants, the urbanisation level was assigned – predominantly urban area, medially urban area and predominantly rural area – following the classification proposed by the Portuguese National Institute of Statistics^(32)^. The education level was recoded into equal or less than 6 years of schooling, 7 to12 years, or more than 12 years. For those aged less than 18 years, the parents’ highest education was considered. Data on household food insecurity were obtained for adults by applying a slightly modified questionnaire developed by Bickel *et al.*^(33)^, as previously described^(22)^. Participants with moderate insecurity were combined with those with severe insecurity in order to analyse food security *v.* food insecurity.

Physical activity was accessed by the International Physical Activity Questionnaire (IPAQ) short-form^(34)^ in those aged 15 and above. Each participant was classified as active, minimally active or inactive.

In terms of smoking habit, each participant aged 15 or over was rated for tobacco use. Three classes were used: ‘never smoked’, ‘former smoker’ (those who smoked but currently no longer smoke) and ‘current smoker’. In the case of adolescents, only information was collected on whether they have never smoked or currently smoking, being not possible to identify former smokers.

### Statistical analysis

Descriptive data of the absolute usual intake of UPF and non-UPF (in quantity – g) as well as of the relative contribution of UPF to the total quantity of food (% TQ) were studied through the analysis of its distribution. The usual intake distributions were obtained using the Statistical Program to Assess Dietary Exposure (SPADE) software^(35)^. Briefly, this software applies the following steps: (1) Box–Cox transformation (transforming observed food consumption results into a symmetrical distribution); (2) modelling daily food consumption as a function of age (using fractional polynomials in order to estimate intra- and inter-individual variance using a linear random-effects model); (3) re-transformation to the original scale (based on solving an integral using the Gaussian quadrature method and the parameter estimates of the previous step).

Probabilistic weights were used to achieve representativeness of the Portuguese population, in order to compensate for oversampling of regions and age groups. Moreover, the use of the probabilistic weights accounts for the cluster effect of primary sample units (primary health care units) and the stratification by the region (NUT II).

In the study of associated factors, UPFs were considered as absolute quantity instead of a proportion for the following reasons. First, when estimating a proportion of the quantity of UPF to the total quantity of foods consumed, it is assumed that UPF and total food consumption are directly proportional on a logarithmic scale (Supplementary Figure S1). This mathematical assumption was checked, and it was noticed that, in this case, the consumption of UPF grows as a function of the square root of the total quantity of food (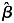: 0⋅486, 95 % CI: 0⋅364, 0⋅607). Second, the predictive capacity of that model proved to be low (*R*^2^ 0⋅009) that is, even correcting the power of the denominator, the total quantity explains only 0⋅9 % of the UPF quantity variance. In addition, we found weak correlations between the quantity of UPF and the total food quantity (*ρ*  0⋅16) and between the quantity of UPF and the quantity of non-UPF (*ρ* −0⋅17). It should be noted that other authors^(36)^ already warned that choosing relative approaches may create mathematical dependency between the numerator and the denominator, introducing residual confounding.

Linear associations between sociodemographic and behavioural factors and the quantity of UPF consumption based on 2-d mean intake were evaluated through linear regression coefficients (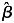) and the respective 95 % CI. The 2-d mean intake is more symmetric than the usual mean in which the 2 days for each individual are used. The residuals of the models showed a reasonable skewness, around +1⋅5, which is acceptable for large samples, as it is between −2 and +2^(37)^. In addition, the histogram and the Q–Q graph were checked, with a small deviation being observed, which allowed for the use of linear regression models.

According to the literature, sex seems to have a modifying effect on several of the studied exposures. Therefore, the effect of sex on the relationship of each of the other variables studied with the consumption of UPF was evaluated and several significant results were found (*P* < 0⋅001). For this reason, the analysis of associated factors was stratified by sex. The modifying effects were tested using nested models with and without interaction, observing the significance of the interaction using ANOVA with Rao-Scott LRT. In addition to the crude model, an adjusted model for age group and education level (Model 1) was assessed, as well as a model with an additional adjustment for the remaining quantity of foods consumed (non-UPF) (Model 2).

A significance level of 0⋅05 was considered. Statistical analyses were carried out using R software^(38)^, version 3⋅6⋅1 for *Windows*. Probabilistic weights were applied using the package ‘survey’^(39)^, according to the complex sampling design.

## Results

Table 1 shows the main characteristics of the sample and describes its UPF consumption. In the Portuguese population aged between 3 and 84 years, the median UPF consumption was 257 g/d, which corresponds to 10⋅6 %TQ (454 kcal/d; 23⋅8 of the total energy intake (%TEI) – data not shown). The absolute quantity consumed seems to be higher in males (263 *v*. 254 g/d in females), although the relative consumption to the total food quantity was higher in females (11⋅4 *v*. 9⋅9 %TQ in males). Regarding the age groups, the absolute consumed quantity seems to be higher in adolescents, reaching 490 g/d, while analysing the relative contribution it seems to be higher in children, tending to decrease with increasing age (children: 22⋅3 %TQ; adolescents: 21⋅6 %TQ; younger adults: 13⋅4 %TQ; adults: 7⋅9 %TQ; elderly: 5⋅4 %TQ). For the geographical regions, the UPF consumption seems to be higher in the Autonomous Region of Azores (an archipelago) and in the Metropolitan Area of Lisbon (capital), counting on 300 and 296 g/d, respectively. It should also be noted that the Autonomous Region of Madeira showed to have the lowest absolute UPF consumption (237 g/d). However, as it also presented one of the lowest non-UFF consumptions, the contribution of UPF to the total quantity of food consumed was higher, which reinforces the use of the absolute quantity as a measure of UPF assessment to avoid residual confounding. Individuals with less than 6 years of education showed the lowest UPF consumption (209 g/d) and also the lowest non-UPF consumption (2075 g/d). Higher educated participants were those with the smallest UPF contribution to the total quantity since they consume more UPF but also more non-UPF. Higher UPF consumption was observed for current smokers (274 g/d *v*. never smoked: 231 g/d; former smoker: 236 g/d). However, former smokers were those who consumed more non-UPF (never smoked: 2124 g/d *v*. former smokers: 2393 g/d *v*. current smoker: 2257 g/d). For the remaining studied variables, no appreciable differences were found for descriptive purposes.

**Table 1.** Ultra-processed foods usual consumption according to sociodemographic and behavioural characteristics (weighted for the distribution of the Portuguese population)

	*n*	Weighted %	Quantity of UPF (g)	Quantity of non-UPF (g)	% of UPF to total quantity (g / g)
			Median (P25–P75)	Median (P25–P75)	Median (P25–P75)
	5005	100	257 (141–426)	2176 (1752–2645)	10⋅6 (5⋅9–17⋅7)
Sex					
Female	2613	51⋅2	254 (151–393)	1986 (1610–2392)	11⋅4 (6⋅8–18⋅0)
Male	2392	48⋅8	263 (133–462)	2412 (1962–2903)	9⋅9 (5⋅0–17⋅5)
Age group					
Children (3–9 years)	521	5⋅9	414 (273–595)	1439 (1129–1798)	22⋅3 (15⋅5–30⋅8)
Adolescents (10–17 years)	632	8⋅1	490 (337–684)	1841 (1484–2247)	21⋅6 (15⋅0–30⋅0)
Younger adults (18–44 years)	1758	38⋅3	340 (217–503)	2244 (1838–2701)	13⋅4 (8⋅5–19⋅9)
Older adults (45–64 years)	1344	29⋅7	195 (115–306)	2313 (1904–2773)	7⋅9 (4⋅7–12⋅3)
Elderly (65–84 years)	750	17⋅9	120 (66–201)	2171 (1776–2616)	5⋅4 (3⋅0–8⋅9)
Education					
≤6 years	1497	29⋅7	209 (104–389)	2075 (1668–2534)	9⋅5 (4⋅7–17⋅9)
7–12 years	2201	45⋅8	284 (165–448)	2221 (1794–2690)	11⋅5 (6⋅7–18⋅4)
>12 years	1291	24⋅5	284 (174–428)	2202 (1791–2655)	11⋅5 (7⋅0–17⋅5)
Region					
North	838	35⋅4	242 (129–409)	2137 (1728–2589)	10⋅2 (5⋅5–17⋅3)
Centre	886	21⋅8	249 (143–400)	2191 (1769–2659)	10⋅2 (5⋅9–16⋅7)
Lisbon Metropolitan Area	701	26⋅8	296 (167–477)	2209 (1778–2678)	12⋅0 (6⋅9–19⋅3)
Alentejo	575	6⋅6	268 (147–447)	2319 (1808–2894)	10⋅5 (5⋅7–17⋅8)
Algarve	654	4⋅3	281 (161–445)	2401 (1955–2882)	10⋅4 (5⋅8–17⋅1)
Autonomous Region of Madeira	683	2⋅7	237 (128–396)	1962 (1604–2362)	11⋅5 (6⋅3–19⋅1)
Autonomous Region of Azores	668	2⋅5	300 (174–475)	1893 (1493–2346)	13⋅7 (8⋅0–21⋅6)
Urbanisation level					
Predominantly urban area	3650	77⋅6	262 (144–431)	2167 (1751–2625)	10⋅8 (6⋅0–17⋅9)
Medially urban area	863	13⋅8	245 (133–412)	2182 (1719–2711)	9⋅9 (5⋅3–17⋅1)
Predominantly rural area	492	8⋅5	247 (134–414)	2172 (1740–2648)	10⋅2 (5⋅6–17⋅0)
Civil status					
Single, divorced or widowed	1495	394	243 (137–398)	2234 (1802–2717)	9⋅9 (5⋅8–15⋅9)
Married, couples	2354	606	225 (118–388)	2283 (1880–2735)	9⋅0 (4⋅9–15⋅1)
Household members					
1–2	1639	39⋅9	266 (140–456)	2181 (1740–2659)	10⋅8 (5⋅8–18⋅2)
3–4	2752	54⋅8	255 (135–425)	2144 (1724–2613)	10⋅5 (5⋅7–17⋅6)
≥5	468	8⋅3	232 (124–393)	2075 (1668–2534)	9⋅8 (5⋅0–16⋅9)
Food insecurity					
No	3448	89⋅9	236 (128–396)	2277 (1868–2734)	9⋅4 (5⋅3–15⋅5)
Yes	397	10⋅1	204 (102–372)	2283 (1880–2735)	9⋅4 (4⋅8–16⋅6)
Physical activity level					
Inactive	1693	43⋅2	242 (130–412)	2142 (1759–2563)	10⋅2 (5⋅6–17⋅0)
Minimally active	1233	30⋅3	234 (130–387)	2240 (1825–2695)	9⋅6 (5⋅4–15⋅6)
Active	1022	6⋅5	236 (124–405)	2375 (1925–2887)	9⋅0 (4⋅9–15⋅0)
Smoking status					
Never smoked	1952	49⋅4	231 (123–391)	2124 (1734–2555)	9⋅8 (5⋅4–16⋅1)
Former smoker	1157	30⋅1	236 (126–402)	2393 (1948–2892)	9⋅0 (4⋅8–15⋅3)
Current smoker	831	20⋅5	274 (151–460)	2257 (1836–2729)	11⋅1 (6⋅4–18⋅2)

UPF, ultra-processed foods.

Table 2 shows the association between sociodemographic and behavioural characteristics and the absolute quantity of UPF by sex. After adjustment for education and non-UPF consumption (Model 2), considering older adults as a reference, all lower age groups were positively associated with UPF consumption in males and females. The magnitude was higher in adolescents and different by sex (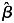 females: 192, 95 % CI: 135, 249; 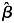 males: 327, 95 % CI: 277, 377). Differences by sex were also observed in younger adults (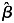 females: 100, 95 % CI: 87, 133; 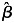 males: 235, 95 % CI: 190, 280). Elderly individuals showed a significantly lower consumption of UPF than adults (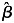 females: −63, 95 % CI: −91, −34; 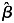 male: −51, 95 % CI: −93, −9). In both sex, the lowest level of education was associated with a lower consumption of UPF when compared to the highest level (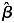 females: −51, 95 % CI: −86, −16; 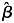 males: −68, 95 % CI: −124, −12). A sensitivity analysis stratifying by age group (Supplementary Table S1) confirmed that this inverse association was only observed in adults (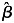 younger adults: −117; 95 % CI: −197, −37; 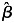 older adults: −75; 95 % CI: −115, −34) and in children a lower level of education was associated with high consumption of UPF (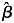 children: 111; 95 % CI: 13, 209). Furthermore, no significant association was observed in adolescent and elderly groups. Only in males, the consumption of UPF among those who were married/couples was lower when compared to singles (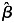: −48, 95 % CI: −96, −1). In females, being a current smoker or a former smoker was associated with a higher consumption of UPF compared to those who reported never having smoked (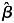 current smoker: 79, 95 % CI: 41, 118; 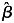 former smoker: 42, 95 % CI: 8, 75).

**Table 2.** Association between sociodemographic and behavioural characteristics and ultra-processed foods usual consumption (g), stratified by sex (weighted for the distribution of the Portuguese population), using linear regression models

	Females			Males		
	Crude model 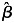 (95 % CI)	Model 1 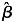 (95 % CI)	Model 2 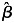 (95 % CI)	Crude model 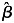 (95 % CI)	Model 1 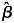 (95 % CI)	Model 2 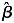 (95 % CI)
Age group						
Children (3–9 years)	195 (148, 242)	179 (130, 228)	140 (89, 191)	**241** (**195, 287)**	**221** (**174, 269)**	**179** (**128, 231)**
Adolescents (10–17 years)	228 (174, 282)	215 (156, 274)	192 (135, 249)	**367** (**317, 418)**	**348** (**298, 398)**	**327** (**277, 377)**
Younger adults (18–44 years)	119 (88, 149)	103 (70, 137)	100 (67, 133)	**260** (**215, 305)**	**236** (**191, 282)**	**235** (**190, 280)**
Older adults (45–64 years)	Ref.	Ref.	Ref.	Ref.	Ref.	Ref.
Elderly (65–84 years)	−80 (−109, −51)	−64 (−93, −35)	−63 (−91, −34)	−**62** (−**100,** −**23)**	−41 (−82, 1)	−**51** (−**93,** −**9)**
Region						
North	Ref.	Ref.	Ref.	Ref.	Ref.	Ref.
Centre	−4 (−35, 28)	4 (−28, 35)	7 (−26, 40)	−22 (−72, 29)	−4 (−53, 45)	0 (−51, 52)
Lisbon Metropolitan Area	47 (7, 87)	39 (−1, 80)	39 (−3, 81)	**85** (**24, 146)**	**70** (**17, 124)**	**76** (**19, 133)**
Alentejo	34 (−10, 77)	46 (9, 83)	50 (9, 90)	−1 (−65, 63)	30 (−32, 91)	41 (−23, 106)
Algarve	34 (−4, 72)	27 (−5, 60)	36 (1, 70)	21 (−26, 68)	23 (−22, 68)	32 (−17, 80)
Autonomous Region of Madeira	−14 (−44, 15)	−13 (−46, 20)	−23 (−59, 13)	21 (−33, 75)	5 (−36, 46)	−7 (−53, 39)
Autonomous Region of Azores	62 (15, 110)	50 (7, 93)	40 (−3, 90)	**112** (**29, 194)**	91 (−12, 171)	82 (−3, 167)
Education						
≤6 years	−136 (−166, −105)	−38 (−74, −2)	−51 (−86, −16)	−**200** (−**251,** −**148)**	−**64** (−**120,** −**9)**	−**68** (−**124,** −**12)**
7–12 years	11 (−18, 39)	26 (−1, 54)	21 (−6, 49)	9 (−35, 53)	12 (−27, 52)	7 (−32, 46)
>12 years	Ref.	Ref.	Ref.	Ref.	Ref.	Ref.
Urbanisation level						
Predominantly urban area	Ref.	Ref.	Ref.	Ref.	Ref.	Ref.
Medially urban area	−10 (−40, 19)	−12 (−46, 23)	−12 (−49, 24)	−17 (−71, 37)	−2 (−47, 44)	1 (−65, 67)
Predominantly rural area	−16 (−63, 32)	−21 (−63, 20)	−21 (−61, 20)	−33 (−92, 26)	−2 (−64, 60)	0 (−47, 48)
Civil status						
Single, divorced or widowed	Ref.	Ref.	Ref.	Ref.	Ref.	Ref.
Married, couples	−34 (−64, -3)	−14 (−42, 13)	−10 (−38, 17)	−**144** (−**195,** −**92)**	−**50** (−**98,** −**3)**	−**48** (−**96,** −**1)**
Household members						
1–2	Ref.	Ref.	Ref.	Ref.	Ref.	Ref.
3–4	94 (64, 124)	−7 (−37, 24)	−6 (−37, 25)	**138** (**95, 181)**	15 (−26, 56)	13 (−29, 54)
≥5	72 (22, 122)	−23 (−75, 28)	−25 (−79, 29)	**178** (**101, 254)**	7 (−63, 78)	7 (−63, 77)
Food insecurity						
No	Ref.	Ref.	Ref.	Ref.	Ref.	Ref.
Yes	−36 (−74, 3)	−7 (−41, 27)	−11 (−43, 22)	−64 (−145, 16)	−32 (−100, 35)	−43 (−109, 23)
Physical activity level						
Inactive	Ref.	Ref.	Ref.	Ref.	Ref.	Ref.
Minimally active	−25 (−58, 8)	−26 (−57, 4)	−24 (−55, 6)	−13 (−59, 32)	−17 (−58, 23)	−11 (−52, 30)
Active	0 (−40, 40)	0 (−35, 35)	5 (−31, 40)	47 (−10, 104)	−5 (−57, 46)	6 (−48, 60)
Smoking status						
Never smoked	Ref.	Ref.	Ref.	Ref.	Ref.	Ref.
Former smoker	68 (35, 102)	35 (2, 68)	42 (8, 75)	−**58** (−**106, −11)**	7 (−32, 46)	12 (−27, 51)
Current smoker	128 (82, 174)	83 (45, 121)	79 (41, 118)	−14 (−68, 40)	−12 (−61, 37)	−11 (−60, 39)

Model 1, adjusted for age group and education, Model 2, adjusted for age group, education and non-ultra-processed foods consumption.

Bold denotes statistical significance (p-value <0.05).

**Fig. 1.**
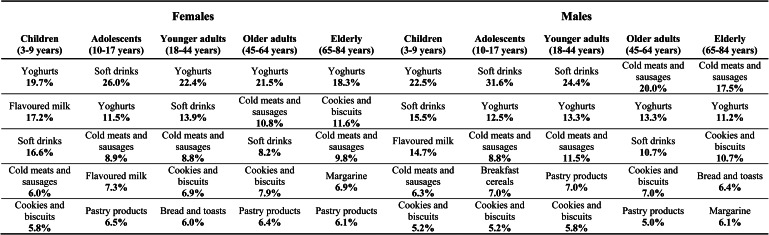
Top contributing food groups to the consumption of ultra-processed foods (UPF) (quantity of UPF from each food group (g) divided by the total quantity of UPF (g)), by age group, stratified by sex (weighted for the distribution of the Portuguese population).

**Fig. 2.**
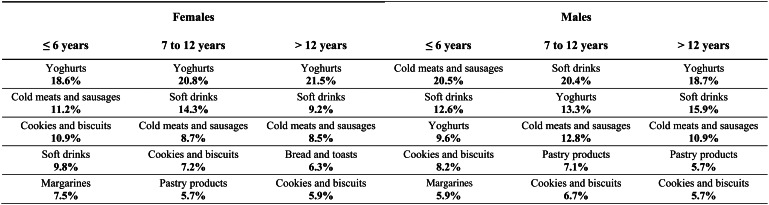
Top contributing food groups to the consumption of ultra-processed foods (UPF) (quantity of UPF from each food group (g) divided by the total quantity of UPF (g)), by education level, stratified by sex (weighted for the distribution of the Portuguese population).

The top contributing food groups to the total quantity of UPF are presented in Fig. 1, for the different age groups, stratified by sex. Overall, in females, the leading contributor to the UPF quantity was yoghurts and other fermented milks containing cosmetic additives hereinafter described only as yoghurts (20⋅3 %) and, in males, soft drinks (17⋅2 %) (data not shown). In both sex, children were the age group in which the three highest food contributors (yoghurts, flavoured milks and soft drinks) had more similar magnitude between them. In females, yoghurts showed the highest contribution in all age groups with the exception of adolescents, in which soft drinks led and whose difference to the second contributor (yoghurts) is notorious (26⋅0 *v*. 11⋅5 %, respectively). This gap between the first and second contributors was also observed in adolescent males (31⋅6 and 12⋅5 %, respectively). Soft drinks were still the main contributor to UPF consumption in young adult males (24⋅4 %). Cold meats and sausages take on greater importance in the oldest age groups of males (adults: 20⋅0 %; elderly: 17⋅5 %). Only in the elderly, soft drinks were not among the main UPF contributors. On the other hand, the food group of cookies and biscuits emerged as a top contributor (females: 11⋅6 %; males: 10⋅7 %). Likewise, in Fig. 2, the top contributing food groups to the consumption of UPF are presented for the different education levels, stratified by sex. In females, regardless of education level, the main food contributor to the UPF was yoghurts. This food group deserves to be highlighted in the upper class since the contribution of the other groups was much lower (21⋅5 % *v*. soft drinks: 9⋅2 % and cold meats and sausages: 8⋅5 %). Cookies and biscuits showed a relevant contribution only in females with a lower level of education (10⋅9 %). On the other hand, in males, greater heterogeneity was observed in the food groups that mostly contributed to the consumption of UPF, according to the class of education. The contribution of yoghurts increased with the increase in schooling (from 9⋅6 to 18⋅7 %), in contrast to that of cold meats and sausages, which tends to decrease (from 20⋅5 to 10⋅9 %). The contribution of soft drinks was higher in the intermediate class (20⋅4 % *v*. ≤6 years: 12⋅6 %; >12 years: 15⋅9 %).

## Discussion

The present study found an UPF consumption of 257 g/d (10⋅6 %TQ; 454 kcal/d; 23⋅8 %TEI), using data from 2015/2016 of the Portuguese population aged between 3 and 84 years. In addition, we highlight the relevant role of sex, age and educational level, since these were the factors with a higher association with UPF consumption, influencing their food sources. The purchase of UPF in Portugal was estimated to provide 10 % of the household total available dietary energy in the year 2000^(40)^. An increase in UPF consumption from 2000 to 2015/2016 may have occurred. However, the results of these studies cannot be directly compared since data from 2000 refer to household data, whereas the 2015/2016 data came from individual dietary survey. As such, Household Budget Surveys have some limitations with respect to individual consumption data, notably foods wasted and foods eaten while dining out, which were not accounted for. UPF consumption was lower in Portugal than in most countries where it has been studied for similar age groups. Data from European countries namely from the Belgian Food Consumption Survey 2014/2015 showed that UPF contribute 32⋅6 % to total energy intake,^(41)^ while data from the UK National Diet and Nutrition Survey 2008–2014 pointed for 56⋅8 %^(8)^. One of the possible justifications for the lower consumption in Portugal could be the adherence to traditional dietary patterns such as the Mediterranean Diet^(42,43)^ or the Southern European Atlantic Diet^(44)^. Recently, a study in the neighbouring country Spain showed that adherence to the traditional Mediterranean Diet was inversely associated with energy intake from UPF^(45)^. Portugal is a country that still preserves traditional eating habits based on fresh and minimally processed foods and culinary preparations made with these foods^(42)^. In Spain, UPF accounted to 24⋅4 %TEI among adults corresponding to 385 kcal/d^(46)^. Despite the differences in age groups between our study and the Spanish study, it is interesting to highlight that although the proportion in Spain is slightly higher than in Portugal (24⋅4 *v*. 23⋅8 %TEI), the absolute energy intake from UPF estimates is lower (385 *v*. 454 kcal/d), which suggests the need for a careful interpretation of the results when the proportion of TEI is expressed. We have also estimated the proportion of UPF to the total amount of food in order to take into account UPF that provides low or no energy (e.g. artificially sweetened beverages). Other previous studies have already analysed the consumption of UPF using the quantity proportion in order to account for low or no caloric food products. A study conducted on French adults (NutriNet-Santé)^(47)^ found a value lightly higher than ours (17⋅4 *v*. 10⋅6 %TQ). This was an online study using convenience sampling from the general population, which may justify the differences in these geographically close countries.

In the present study, younger ages and higher education levels were significantly associated with higher UPF consumption. Due to the cross-sectional nature of this study, it is not possible to assess a potential cohort effect. However, it is expected that the younger people from the current population could maintain these high levels in the future. These ideas are supported by tracking the effect of food patterns previously described during childhood^(48,49)^ and from adolescence into early adulthood^(50,51)^. Similar results were found in Mexico in 2012 where younger ages and medium and high socio-economic status were also related to higher UPF consumption^(52)^. In addition, urbanisation and living in the North of Mexico were also sociodemographic factors related to higher consumption of UPF. Similar findings were reported in Colombia^(18)^. On the other hand, in the USA, the UPF consumption decrease with the income level and with the level of education^(53)^. As countries become wealthy, its middle class will develop and tend to consume more UPF, typically produced by big multinational brands, maybe to exhibit socio-economic status^(1)^. Moreover, in the present study, the different effects of the education level on UPF consumption observed by age can portray the nutritional transition over the generations. A study on the evolution of UPF prices over time in Brazil^(54)^ found that in 1995, UPFs were the most expensive food group. Since the early 2000s, prices of UPF underwent successive reductions, becoming cheaper and predictions point that in 2026 they will be even cheaper than minimally processed foods and culinary ingredients. With a lack of education in this matter, it is quite easy to be misled and to yield towards the marketing pressure. In Scotland, differences in exposure to food advertisements across areas with different socio-economic positions were observed, with higher exposures in low socio-economic areas^(55)^. In the present study, being a single male or a smoking female was shown to be associated with higher UPF consumption. Blanco-Rojo *et al.* reported a positive association between current smoking and UPF consumption in Spain^(46)^, as well as Rauber *et al.*, in UK adults^(20)^. This positive association was also observed in the present study, suggesting an aggregation of these behaviours. Time doing sedentary activities (watching TV not included) and higher activity index were also associated with UPF consumption in Spain, as in the study of Rauber *et al.*, but not in the present study. These differences can be explained by the use of different physical activity assessment methodologies and their respective categorisation as well as by a possible higher consumption of non-UPF in the higher classes of physical activity. Marital status has been less studied in this regard. In males, we found an inverse association between being married/couples and the consumption of UPF. We believe that this is due to eating more meals at home based on culinary preparations. In UK adults, better home food preparation skills and more frequent use of these skills tended to be associated with a lower UPF consumption^(56)^.

The present study was able to show that the top food groups contributing to UPF consumption were different by sex and according to the age group and education. In general, Portuguese individuals with a higher education level consumed higher quantity of UPF, with yoghurts being the main source, both in females and males. In the lower level of education, particularly in males, cold meats and sausages and soft drinks take on relevance. As the level of education increases, the consumption of cold meats and sausages decreased contrarily to what happened with the consumption of yoghurts. It highlights the need to tailor food programmes and policies to each segment of the population in order to promote its effectiveness. The present study also showed some differences in food sources of UPF consumption compared to other countries. Although the contributions by age group were not described, in general, in the UK^(8)^ and in Canada^(57)^, industrialised packaged breads seem to be the highest contributor of UPF consumption. In Portugal, this was not expected because traditionally Portuguese people buy fresh bread at bakeries (considered as processed by the NOVA classification – NOVA group 3). In the Belgian national food consumption survey 2014–2015^(41)^, milk beverages were the dominated source of UPF among children aged 3–5 years followed by cold meats and sausages. In the aforementioned study, except for children under 5 years old and for girls aged 14–17, cold meats and sausages were the top contributor. Whereas cakes, pies, pastries and the dry cakes and sweet biscuits, and soft drinks were shown to be relevant contributions, mostly in males and younger ages. In Portuguese children, yoghurts, flavoured milks and soft drinks went hand in hand, both having relevant contributions. In adolescents, who consume the highest absolute quantity of UPF, the biggest concern seemed to be the soft drinks.

The differences by sex were greater in adults, where yoghurts were the main contributor in females and soft drinks in males. Dairy products are common in Portugal probably due to greater adherence to the Mediterranean diet compared to other countries. However, over the past 100 years, continuous development and improvement from the ingredients selection to the technological processing techniques brought a huge variety of yoghurts to the market^(58)^. Due to the inherent diversity in the nutrient contents and food matrices of different dairy products, the association between dairy and disease risk has often been contradictory^(59)^. In the present study, unsweetened plain yoghurts were classified as minimally processed foods, plain yoghurts with added sugar as processed and flavoured yoghurts or with artificially sweeteners as ultra-processed. Focus group studies have made it possible to understand the population's perception of UPF^(60)^. In a socio-economically diverse sample from Uruguay, these products were not perceived as harmful to health. Some categories of UPFs were classified as unhealthy (e.g. mayonnaise, potato chips), while others tended to be classified as healthy (e.g. yogurt, granola). For this reason, we suggest food interventions to be based on holistic perspective of foods, promoting food literacy that encompasses nutritional composition and ingredients and thus promoting the consumption of minimally processed yoghurts instead of the UPF alternatives. The industrial reformulation of these yoghurts in order to reduce the use of food additives should also be encouraged. As an attempt to do this to soft drinks production/consumption, in 2017, Portugal introduced a special tax^(61,62)^. Preliminary results of the first year after the tax implementation showed that the industry responded with a reformulation of the products^(63)^. However, the substitution of sugar by additives with a sweetening effect deserves attention^(64)^. Despite different contributors to UPF in different populations, it cannot be directly hypothesised that the health effects may be different because of this, so further studies are encouraged to clarify these relations.

There were several strengths and limitations in the present study. Firstly, data from a national representative sample were used in this study, ensuring external validity. In addition, the interviews were carried out by highly trained nutritionists according to standardised procedures and using computer-assisted personal interviewing. Data inclusion was easier, systematic and accurate due to the use of the electronic platform, specifically designed for the present project, but following harmonised European procedures^(24)^. Food records and 24 h recalls methods were listed as those with the best performance in estimating UPF (‘high to very high’ and ‘very high’ potential, respectively) as they allow to obtain a high level of detail for each food^(65)^. The multiple-pass dietary interviews minimised the omission of possible forgotten foods. Moreover, this method standardised the level of detail for the description of foods and their quantification, including the portion size estimation by photographs of different portions. Seasonal variability was accounted for since the fieldwork was carried out within 12 months. Dietary assessment included 2 d, evenly distributed through the week^(23)^, despite knowing that non-healthy food consumption appears to be higher on weekends^(66)^. However, an adequate distribution of reports corresponding to weekdays and weekend days was obtained. Still, to give robustness to our estimates, usual intake was modelled using SPADE, in which intra-individual variation is estimated and eliminated.

The use of NOVA classification, widely assumed as a proper classification system based on food processing, also values this work. In general, the studies on UPF consumption and its associated factors currently available use the energy proportion as the outcome. Instead, in this study, the absolute quantity was used whereby comparability with other studies may be affected. However, in order to minimise this limitation, descriptive results on UPF consumption were presented as the relative contribution of UPF both to total energy intake and to total quantity of food. The use of the absolute quantity gives strength to the results since it gives relevance to low or no calorie foods (which have a considerable consumption in Portugal – from 10⋅6 % of UPF quantity in elderly to 18⋅5 % in adults – data not shown or published) while avoiding the residual confounding effect of dividing by energy. The study of UPF through absolute quantity instead of energy will allow us to perceive changes in the consumption of certain foods that have been taxed in the meantime, such as soft drinks. If only considering the energy contribution of UPF, it can be hypothesised that consumption has decreased when in fact it may have been maintained or increased but masked by the use of artificial sweeteners.

## Conclusion

In the Portuguese population aged between 3 and 84 years, the UPF consumption was 313 g/d (10⋅6 %TQ; 454 kcal/d; 23⋅8 %TEI). Compared to older adults, younger ages were significantly associated with a higher consumption of UPF in both sexes. In adolescents and younger adults, males consumed more UPF than females. Higher education was shown to be positively associated with UPF consumption in adults and negatively in children. Being a single male or a smoking female were other factors that were positively associated with high UPF consumption. Overall, yoghurt-containing additives, soft drinks and cold meats and sausages were the highest contributors to UPF quantity. Yoghurts showed to be the leaders for children and adult females at all levels of education while soft drinks showed to be the leaders in adolescents from both sexes and in younger adults. Cold meats and sausage consumption stood out in males with lower educational levels, decreasing as schooling increases, as opposed to yoghurt consumption. The differences found in these contributors by age group and educational level deserve to be taken into account when designing effective interventions aiming to decrease UPF consumption. However, for some foods such as yoghurts, emphasis should be placed on promoting consumption of the minimally processed ones.

## Supplementary material

For supplementary material accompanying this paper visit http://dx.doi.org/10.1017/jns.2021.61. click here to view supplementary material
