# Supplementary material for: Associated factors to the consumption of ultra-processed foods and its relation with dietary sources in Portugal
Source: J Nutr Sci. 2021 Oct 7;10:e89. doi: 10.1017/jns.2021.61 (PMC8532074; doi:10.1017/jns.2021.61)
Supplement: Supplementary file 1 [file S2048679021000616sup001.docx]

Supplemental table 1. Association between sociodemographic and behavioural characteristics and ultra-processed foods usual consumption (grams), stratified by age group (weighted for the distribution of the Portuguese population), using linear regression models.

|  | **Children (3-9 years)** | | **Adolescents (10-17 years)** | | **Younger adults (18-44 years)** | | **Older adults (45-64 years)** | | **Elderly (65-84 years)** | |
| --- | --- | --- | --- | --- | --- | --- | --- | --- | --- | --- |
|  | **Crude model** | **Model 2** | **Crude model** | **Model 2** | **Crude model** | **Model 2** | **Crude model** | **Model 2** | **Crude model** | **Model 2** |
|  | $\hat{\beta}$ (95% CI) | $\hat{\beta}$ (95% CI) | $\hat{\beta}$ (95% CI**)** | $\hat{\beta}$ (95% CI) | $\hat{\beta}$ (95% CI) | $\hat{\beta}$ (95% CI) | $\hat{\beta}$ (95% CI) | $\hat{\beta}$ (95% CI) | $\hat{\beta}$ (95% CI) | $\hat{\beta}$ (95% CI) |
| **SEX** |  |  |  |  |  |  |  |  |  |  |
| Female | Ref. | Ref. | Ref. | Ref. | Ref. | Ref. | Ref. | Ref. | Ref. | Ref. |
| Male | 25 (-32;81) | 36 (-17;88) | 118 (54;182) | 163 (101;225) | 120 (78;162) | 138 (92;183) | -22 (-54;10) | -13 (-45;19) | -3 (-43;37) | 4 (-35;43) |
| **EDUCATION** |  |  |  |  |  |  |  |  |  |  |
| ≤ 6 years | 120 (5;236) | 111 (13;209) | 70 (-35;175) | 24 (-83;131) | -101 (-185;-17) | -117 (-197;-37) | -73 (-113;-33) | -75 (-115;-34) | -72 (-168;24) | -77 (-172;17) |
| 7 to 12 years | 72 (15;130) | 59 (0;118) | 52 (-19;123) | 26 (-45;98) | 35 (-9;80) | 14 (-27;54) | -4 (-41;32) | -4 (-42;33) | -28 (-126;71) | -33 (-131;66) |
| > 12 years | Ref. | Ref. | Ref. | Ref. | Ref. | Ref. | Ref. | Ref. | Ref. | Ref. |
| **REGION** |  |  |  |  |  |  |  |  |  |  |
| North | Ref. | Ref. | Ref. | Ref. | Ref. | Ref. | Ref. | Ref. | Ref. | Ref. |
| Centre | 5 (-49;58) | 28 (-33;88) | -26 (-96;44) | -32 (-96;32) | -11 (-73;51) | -9 (-73;55) | 3 (-48;54) | 2 (-48;52) | 38 (0;75) | 38 (1;76) |
| Lisbon Metropolitan Area | 94 (16;171) | 128 (56;200) | 113 (10;217) | 107 (12;20358) | 41 (-27;109) | 41 (-26;108) | 64 (20;108) | 50 (6;94) | 56 (2;111) | 52 (1;103) |
| Alentejo | 50 (-69;169) | 38 (-84;160) | 88 (-13;190) | 58 (-47;163) | 40 (-24;105) | 57 (-8;122) | 25 (-17;67) | 30 (-13;72) | 44 (-10;99) | 46 (-11;104) |
| Algarve | 84 (-14;181) | 113 (19;207) | 6 (-111;124) | 12 (-82;105) | 7 (-56;70) | 21 (-47;88) | 58 (22;93) | 52 (19;85) | 13 (-18;44) | 17 (-16;50) |
| Autonomous Region of Madeira | -6 (-88;76) | -15 (-106;75) | -63 (-121;5) | -115 (-177;-53) | -27 (-74;20) | -38 (-87;11) | 26 (-3;55) | 27 (-3;57) | 18 (-20;56) | 18 (-22;58) |
| Autonomous Region of Azores | 92 (-31;214) | 60 (-39;91) | 16 (-69;101) | -30 (-121;61) | 77 (19;135) | 66 (-1;132) | 67 (9;124) | 68 (-11;125) | 82 (-5;169) | 72 (-11;156) |
| **URBANIZATION LEVEL** | |  |  |  |  |  |  |  |  |  |
| Predominantly urban area | Ref. | Ref. | Ref. | Ref. | Ref. | Ref. | Ref. | Ref. | Ref. | Ref. |
| Medially urban area | -56 (-116;5) | -70 (-130;-9) | -50 (-115;16) | -53 (-124;18) | 21 (-31;73) | 14 (-39;67) | -32 (-88;23) | -16 (-69;38) | -1 (-54;51) | 7 (-43;58) |
| Predominantly rural area | -77 (-142;-11) | -74 (-162;14) | -39 (-129;50) | -54 (-126;18) | 4 (-90;97) | 7 (-94;109) | -27 (-114;59) | -14 (-97;70) | -15 (-58;28) | 3 (-39;46) |
| **CIVIL STATUS** |  |  |  |  |  |  |  |  |  |  |
| Single, divorced or widowed |  |  |  |  | Ref. | Ref. | Ref. | Ref. | Ref. | Ref. |
| Married, couples |  |  |  |  | -63 (-111;-16) | -40 (-86;6) | -19 (-64;26) | -19 (-64;26) | 1 (-37;38) | 7 (-28;42) |
| **HOUSEHOLD MEMBERS** |  |  |  |  |  |  |  |  |  |  |
| 1 to 2 | Ref. | Ref. | Ref. | Ref. | Ref. | Ref. | Ref. | Ref. | Ref. | Ref. |
| 3 to 4 | -67 (-261;127) | -89 (-269;90) | -42 (-199;116) | -21 (-148;106) | 31 (-18;79) | 27 (-22;76) | 2 (-33;38) | -8 (-43;26) | 0 (-60;60) | -9 (-61;44) |
| ≥ 5 | 26 (-181;233) | -12 (-210;187) | 26 (-150;202) | -1 (-147;145) | -17 (-88;53) | -7 (-79;65) | -38 (-85;10) | -36 (-86;15) | -29 (-108;50) | -28 (-103;47) |
| **FOOD INSECURITY** |  |  |  |  |  |  |  |  |  |  |
| No |  |  |  |  | Ref. | Ref. | Ref. | Ref. | Ref. | Ref. |
| Yes |  |  |  |  | -27 (-93;38) | -9 (-71;52) | -45 (-87;-3) | -34 (-78;11) | -24 (-68;20) | -18 (-61;25) |
| **PHYSICAL ACTIVITY LEVEL** |  |  |  |  |  |  |  |  |  |  |
| Inactive |  |  |  |  | Ref. | Ref. | Ref. | Ref. | Ref. | Ref. |
| Minimally active |  |  |  |  | -44 (-92;4) | -35 (-77;7) | -6 (-51;38) | -4 (-49;40) | 5 (-33;43) | 5 (-36;46) |
| Active |  |  |  |  | 20 (-39;79) | 11 (-46;68) | -18 (-57;22) | -4 (-45;37) | 6 (-47;59) | 19 (-35;74) |
| **SMOKING STATUS** |  |  |  |  |  |  |  |  |  |  |
| Never smoked |  |  |  |  | Ref. | Ref. | Ref. | Ref. | Ref. | Ref. |
| Former smoker |  |  |  |  | 21 (-37;79) | 4 (-51;60) | 44 (14;73) | 53 (22;85) | 35 (-12;82) | 47 (6;87) |
| Current smoker |  |  |  |  | 48 (-3;99) | 28 (-26;81) | 37 (-10;84) | 40 (-6;86) | 27 (-34;88) | 28 (-31;86) |

UPF: ultra-processed foods. Model 2. Adjusted for age group, education and non-UPF consumption.


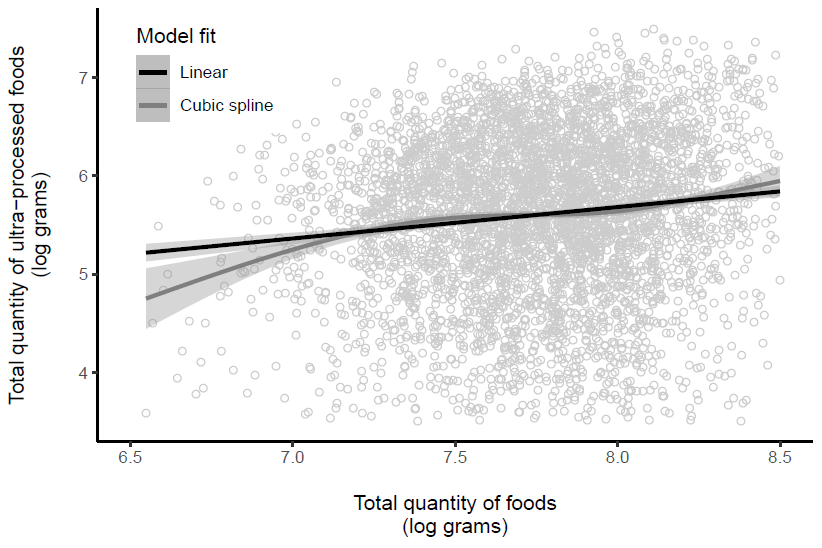


Supplemental figure 1. Association between total quantity of foods and total quantity of ultra-processed foods after logarithmic transformation, applying a linear and cubic spline fit.

This figure supports the decision to study absolute grams instead of their proportion of the total quantity consumed. When modeling the relationship between ultra-processed foods consumption and the total quantity consumed with a cubic spline, we capture the actual trend of the data which shows to be different from the linear relationship, in particular for the lowest and highest values.
